# The Intestinal Efflux Transporter Inhibition Activity of Xanthones from Mangosteen Pericarp: An In Silico, In Vitro and Ex Vivo Approach

**DOI:** 10.3390/molecules25245877

**Published:** 2020-12-11

**Authors:** Panudda Dechwongya, Songpol Limpisood, Nawong Boonnak, Supachoke Mangmool, Mariko Takeda-Morishita, Thitianan Kulsirirat, Pattarawit Rukthong, Korbtham Sathirakul

**Affiliations:** 1 Department of Pharmacy, Faculty of Pharmacy, Mahidol University, Rajathevi, Bangkok 10400, Thailand or panudda_puii@hotmail.com (P.D.); s.limpisood@gmail.com (S.L.); lordrx16@gmail.com (T.K.); 2 Department of Basic Science and Mathematics, Faculty of Science, Thaksin University, Muang Songkhla, Songkhla 90000, Thailand; nawongb@yahoo.com; 3 Department of Pharmacology, Faculty of Pharmacy, Mahidol University, Rajathevi, Bangkok 10400, Thailand; supachoke.man@mahidol.ac.th; 4 Department of Pharmacology, Faculty of Science, Mahidol University, Rajathevi, Bangkok 10400, Thailand; 5 Laboratory of Drug Delivery Systems, Faculty of Pharmaceutical Sciences, Kobe Gakuin University, Kobe 650-8586, Hyogo, Japan; mmtakeda@pharm.kobegakuin.ac.jp; 6 Department of Pharmaceutical Technology, Faculty of Pharmacy, Srinakarinwirot University, Nakornnayok 26120, Thailand; pattarawit@g.swu.ac.th

**Keywords:** xanthones, mangosteen pericarp, P-glycoprotein, caco-2 cell, bidirectional transport, *MDR1* expression, ex vivo absorptive transport

## Abstract

The capacity of α-mangostin (α-MG) and β-mangostin (β-MG) from mangosteen pericarp on P-glycoprotein (Pgp) in silico, in vitro, and ex vivo was investigated in this study. Screening with the ADMET Predictor™ program predicted the two compounds to be both a Pgp inhibitor and Pgp substrate. The compounds tended to interact with Pgp and inhibit Pgp ATPase activity. Additionally, bidirectional transport on Caco-2 cell monolayers demonstrated a significantly lower efflux ratio than that of the control (α-(44.68) and β-(46.08) MG versus the control (66.26); *p* < 0.05) indicating an inhibitory effect on Pgp activity. Test compounds additionally revealed a downregulation of *MDR1* mRNA expression. Moreover, an ex vivo absorptive transport in everted mouse ileum confirmed the previous results that α-MG had a Pgp affinity inhibitor, leading to an increase in absorption of the Pgp substrate in the serosal side. In conclusion, α- and β-MG have the capability to inhibit Pgp and they also alter Pgp expression, which makes them possible candidates for reducing multidrug resistance. Additionally, they influence the bioavailability and transport of Pgp substrate drugs.

## 1. Introduction

Xanthones (α- and β-mangostins) are a significant bioactive compound found in the pericarp of mangosteen (*Garcinia mangostana*). Studies have demonstrated that xanthones contain remarkable pharmacological activities [1,2]. Among xanthones, α-mangostin is considered the most common chemical entity researched so far [3]. Currently, products containing mangosteen juice or extract are experiencing fast growth in markets as dietary supplements, fruit juice, and as a major component in herbal creams, cosmetics, and masks [4]. Numerous studies focus on the pharmacological and pharmacokinetic activity in vitro, yet there are few in vivo studies, particularly the mechanism on intestinal transporters, P-glycoprotein (Pgp) [1,2,3]. Pgp is an ATP-binding cassette (ABC) transporter abundantly found in many organs together with tumor cells. It is a significant protein in cell membranes which actively pumps many foreign substances out of cells, leading to decreased intracellular xenobiotics concentration. Pgp is also known as multidrug resistance protein 1 (MDR1) [5]. Pgp actively transports out a wide variety of chemically diverse compounds. The substrate to Pgp can lead to failure of chemotherapy in cancer patients whose Pgp was upregulated on the surface of the cancer cells [6]. Moreover, binding with Pgp in the intestine can reduce the bioavailabity of compounds [7,8]. Therefore, the recognition of potential Pgp substrates at the early stages of the drug discovery process is essential [9]. In silico, in vitro (in cell cultures), and ex vivo (using inverted intestinal sacs) are the methods to identify and confirm the binding of the substrates to Pgp.

The polarized expression and activity of Pgp in the apical membrane in Caco-2 was evidenced and published by Hunter et al. in 1993 [10]. Thus, the in vitro drug transport experiment in Caco-2 cells has long been utilized to evaluate the effects of molecules on the Pgp transporter. For example, it has been utilized to evaluate the effects of molecules in Japanese kampo medicines on Pgp [11]. The rat everted gut sac model is a useful screening tool to investigate Pgp efflux [12]. This method is an efficient tool for studying the transporter’s role in drug absorption [13]. However, both in vitro and ex vivo experiments are costly and time consuming. Therefore, the application of in silico modelling and simulation within the R&D sector of the pharmaceutical industry is rapidly increasing [14]. The in silico approach can be used from ADMET property prediction. In silico prediction models for Pgp have been developed to give a more accurate prediction of Pgp substrates and Pgp inhibition [9,15,16,17]. The commercially available ADMET Predictor^TM^ program can predict the likelihood of both Pgp efflux and inhibition. It has been recently used in the prediction of the likelihood of natural phytocannabinoids [18].

Previous studies have indicated the capacity of some xanthone scaffolds to alter Pgp [19,20,21]. Recently, Laksmiani [22] found that ethanolic extract of mangosteen pericarp (EEMP) in combination with doxorubicin increased the cytotoxic activity of doxorubicin in MCF-7 cells. The in silico assay by molecular docking with the AutoDock 4.2 program revealed that α-mangostin (as the active compound of EEMP) had an affinity for Pgp with binding energy of −6.13 kcal/mol, and the researcher suggested that an in vitro assay of α-mangostin in inhibiting Pgp activity should be conducted. The objective of this study is to evaluate the effects of xanthones from mangosteen pericarp on Pgp activities in silico, in vitro, and ex vivo.

## 2. Results

### 2.1. In Silico Study via ADMET Predictor^TM^ Software

**Figure 1:**
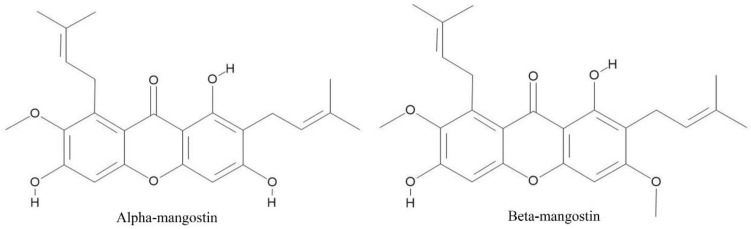
The two-dimensional structure of α-MG and β-MG drawn in the MedChem Designer™ program.

Molecular structures of α- and β-mangostins were drawn two dimensionally via MedChem Designer™ and analyzed via the ADMET Predictor™ program. The structures of the two compounds are xanthone rings with the following functional groups: two double bonds susceptible to hydrogenation, a methoxy group, and hydroxyl groups. The difference between these two compounds is a substitutional group at position 3 of the xanthone ring. The α-mangostins have a hydroxy group, while the β-mangostins have a methoxy group, as shown in Figure 1.

Both mangostins were predicted to be a Pgp inhibitor and Pgp substrate. Probabilities that the prediction was correct for the Pgp substrate in β-MG were the same at 95%, while α-MG was lower (68%). However, the likelihood for the Pgp inhibitor was the same in both xanthone derivatives (97%), as shown in Table 1.

### 2.2. P-Glycoprotein (Pgp) ATPase Assay

The Pgp ATPase assay was employed to screen activity and as a mechanism of the compounds to Pgp ATPase activity via measuring luminescence signals based on Pgp-Glo™ assay systems. The assay measured the ATP remaining in the reaction and interpreted the result from the luminescence signal as relative light units (RLU). The greater the decrease in RLU (remaining ATP), the higher the Pgp activity. Therefore, samples containing compounds that activate the Pgp ATPase will have significantly lower RLU than the untreated sample. However, this assay interpreted an effect of the sample on Pgp ATPase activity (ΔRLU), which was calculated by comparing untreated (NT) samples and samples treated with a test compound (TC) to a Na_3_VO_4_ (sodium orthovanadate)-treated control. Na_3_VO_4_ is a selective inhibitor of Pgp. Consequently, Pgp membranes treated with 100 µM Na_3_VO_4_ had no Pgp ATPase activity. A positive control substrate was 200 µM verapamil, which stimulated Pgp ATPase activity and acted as a competitive Pgp inhibitor [23,24].

**Figure 2:**
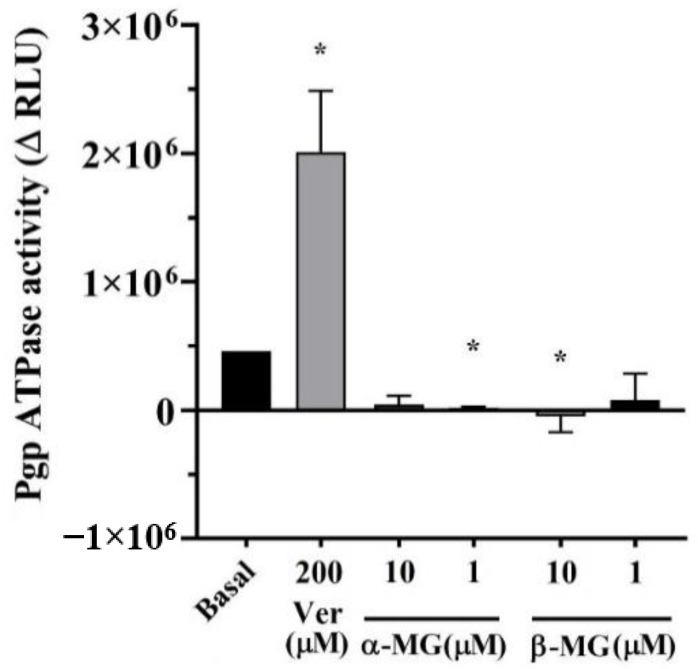
The change in luminescence (ΔRLU) of ATPase assay treated with α-MG and β-MG (*n* = 5) Verapamil (Ver) was used as a positive control substrate. (* *p* < 0.05, compare to basal).

The result of the Pgp ATPase assay was shown as Pgp ATPase activity (ΔRLU), as illustrated in Figure 2. The results revealed that α- and β-MG, at the concentrations of 10 and 1 µM, had lower Pgp ATPase activity than basal. Therefore, both compounds represented the inhibition of Pgp activity.

### 2.3. Cell Culture

#### 2.3.1. In Vitro Cytotoxicity Studies

**Figure 3:**
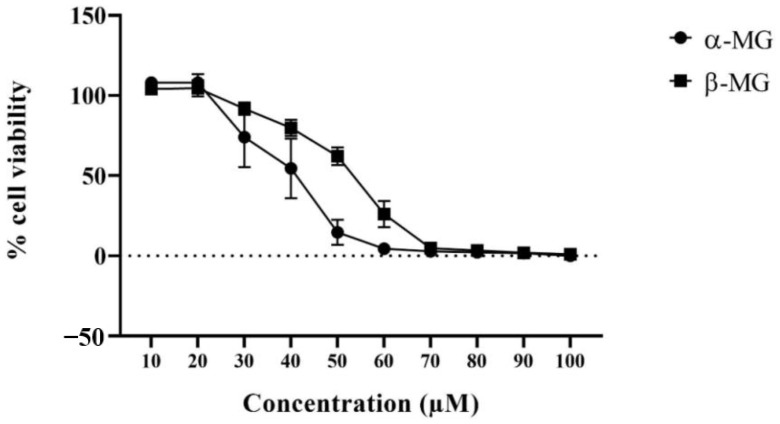
The percent of cell viability treated with the variation of α-MG and β-MG. (*n* = 3).

A cytotoxicity study was used to assess a nontoxic concentration of extracts on Caco-2 cells. The MTT assay results showed that α- and β-MG at concentrations of 10 and 20 µM were not toxic to Caco-2 cells (Figure 3). However, there were few experiments of α-MG at the concentration of 20 µM and β-MG at the concentrations of 10 and 20 µM were below control (91.17% for α-MG, 97.57% and 94.59% for β-MG). Thus, using compounds at a concentration below 10 µM was preferred for future experiments. Half the concentration of the lowest toxic concentration (5 µM) and the same concentration as the Pgp ATPase assay (1 µM) were chosen especially for the transport study to ensure the viability of the cells in all the experiments.

#### 2.3.2. Transport Study

The transport study was a transport of Lucifer Yellow (LY) and Rhodamine 123 (Rho123) across Caco-2 cell monolayers cultured on polycarbonate membrane transwell inserts in 24-well plates at a seeding density of 60,000 cells/cm^2^. The following experiment was started after 20 to 21 days post-seeding. A transepithelial electrical resistance (TEER) value was routinely measured every other day from day 0 to 21. TEER values were used as a sensitive indicator to confirm the integrity, health, and confluence of the cell monolayer before and after the monolayer was evaluated for the transport study.

The transport study was separated into two parts: paracellular transport and bidirectional transport. Moreover, the apparent permeability coefficient (Papp), % Lucifer Yellow (LY) Rejection, and efflux ratio (ER) were calculated. The α- and β-MG were considered as test compounds (TC) for ease of understanding.

##### Paracellular Transport Study

Lucifer yellow, a fluorescent marker, was used as a paracellular maker for evaluating the integrity of Caco-2 cell monolayers during the transport study. The effect of co-solvent (0.5% DMSO) and TC to paracellular transport across the Caco-2 cell monolayers were evaluated by measuring the TEER value, Papp, and %LY Rejection. The TEER value was measured before and after the experiment, while the Papp and LY assays were performed by measuring the passive transport of LY across Caco-2 cell monolayers in the apical-to-basolateral (A–B) direction. Papp for LY of less than 0.4 × 10^−6^ cm/s and %LY Rejection values >99% were reported to be indicative of well-established Caco-2 cell monolayers [25]. The study expressed good formation of a cell monolayer with Papp less than 0.4 × 10^−6^ cm/s; %LY rejection was more than 99% and TEER values illustrated a non-significant difference (*p* > 0.05) between before and after the entire experiment. The findings of this study are TEER values; Papp and %LY Rejection are available in Figure S1 and Table S1 in the Supplementary Materials. Thus, the results showed that adding co-solvent and TC on Caco-2 cells cultured on transwell inserts did not affect the cell monolayers’ integrity and confluence.

##### Bidirectional Transport Study

**Figure 4:**
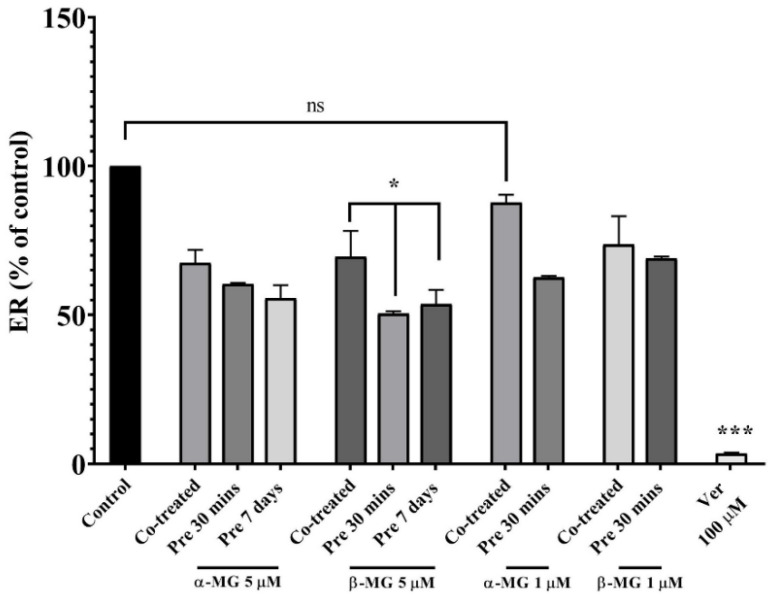
Efflux ratio (ER) showed as a percent of control, Rho123 added simultaneously with test compounds at the beginning (co-treated), at 30 min after incubation with test compounds (pre 30 min), or 7 days after incubation with test compounds (pre 7 days). (*n* = 7) (* *p* < 0.05 compared to the control) (*** *p* < 0.001, compared to the control) (ns; not significant compared to the control).

A bidirectional transport study of Rho123 across Caco-2 cell monolayers was used to evaluate test compounds on Pgp activity under three treatments. The treatments included a co-administration study (co-treated), an inhibition study (pre 30 min), and a 7-day pre-treatment study (pre 7 days). With alteration in Rho123, a Pgp substrate, fluorescence is the marker for interaction of the compound with Pgp. Thus, Rho123 and the test compound in the transport medium were added to a donor compartment. After specific time points, a transport medium from a receiver compartment was sampled. Concentration of Rho123 in the medium was analyzed by a fluorescence microplate reader and efflux activity and the permeability coefficient were calculated. Efflux activity of Pgp was defined as the efflux ratio (ER) and additionally calculated in this study (Table S2). The effects of α- and β-MG on the permeability of Rho123 across Caco-2 cell monolayers were measured as the permeability coefficient (Papp) (Table S2). In all treatment groups, the Papp A–B (an apical-to-basolateral or absorptive transport) of Rho123 was increased, whereas the Papp B–A (a basolateral-to-apical or efflux transport) was significantly decreased, leading to a significant decrease in ER. Only Rho123 co-treated with 1 µM α-MG showed a non-significant difference in ER from the control but a decrease in ER was observed. The 7-day pre-treatment study was used to evaluate the downregulation of Pgp by these compounds. The results also showed the decrease in ER indicated that TC could decrease the amount of Pgp transporter. To be clearly visible in the ER among each extract and treatment group, the ER was calculated as the percent of control (Figure 4). As shown in Figure 4, the co-treated group decreased less ER than other treatments and the significant difference among group treatments was found only in 5 µM β-MG, where the ER of pre 30 min (50.45%) and pre 7 days (53.64%) groups were significantly lower than that of the co-treated group (69.55%).

The results demonstrated an inhibition of Pgp activity or efflux pump activity in the presence of these compounds. In addition, the 5 µM-treated group revealed a higher inhibiting Pgp activity than the 1 µM-treated group as a lower ER.

#### 2.3.3. MDR1 Expression Analysis Using Real-Time RT-qPCR

**Figure 5:**
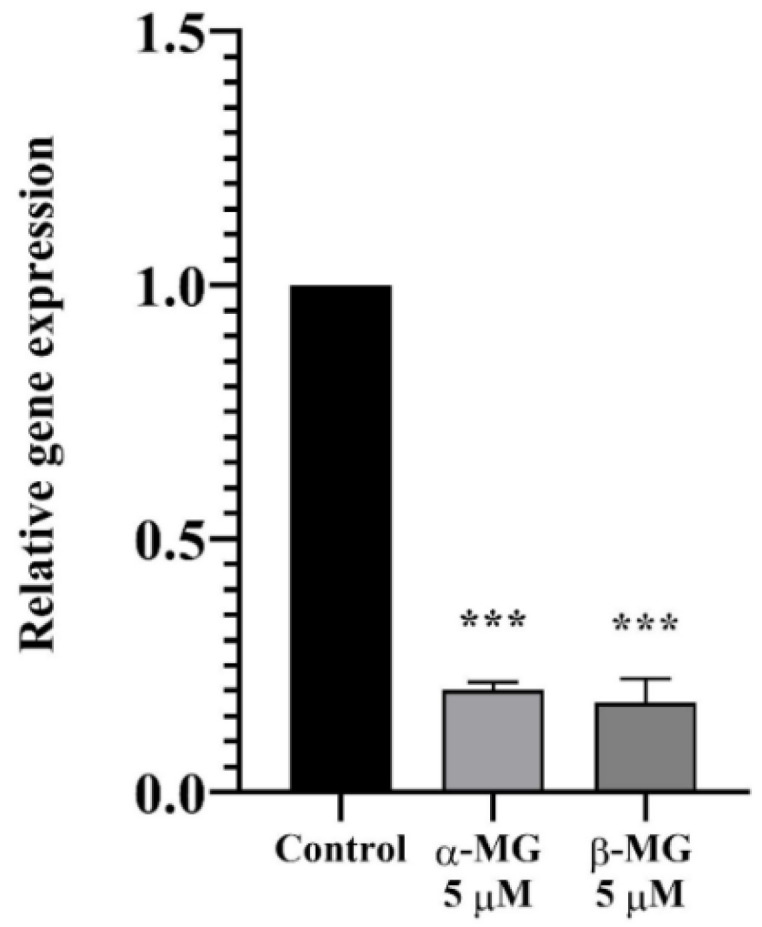
The relative mRNA levels of *ABCB1* gene after being treated with α-MG and β-MG quantified by the real-time RT-PCR. (*n* = 3) (*** *p* < 0.001, compared to the control).

Real-time RT-qPCR was employed to quantify the mRNA levels of the *MDR1* gene after being treated with α- or β-MG at the concentration of 5 µM for 9 h. The relative mRNA levels were calculated and are demonstrated in Figure 5. Both compounds significantly downregulated *MDR1* gene expression (*p* < 0.001), which indicated the decrease in Pgp transporter and its activity.

### 2.4. Ex Vivo Absorptive Transport Study across Everted Mouse Ileum

**Figure 6:**
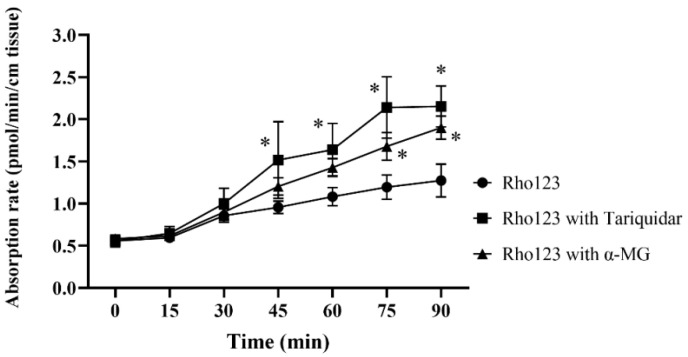
Absorption rate of Rho123 (10 μM) from mucosal to serosal across the everted ileum sac in the presence of tariquidar or α-MG at concentrations 100 nM or 200 μM, respectively. Each value represents the mean ± S.E. * Significantly different from Rho123 (*p* < 0.05).

In the mucosal to serosal study, an ileum was removed from mice, everted, and placed in buffered solution. The transport of Rho123 (a marker for Pgp function) from the mucosal to the serosal side was measured by a microplate fluorescence reader. The absorption rate of Rho123 in the three groups did not show any significant difference at 0 to 30 min. Rho123 administered with Tariquidar (a specific Pgp inhibitor) showed a significant difference in absorption rate from control (Rho123) at times 45 (*p* = 0.0187), 60 (*p* = 0.0198), 75 (*p* = 0.0001), and 90 (*p* = 0.0004) minutes. While Rho123 administered with α-MG showed a significant difference from control at times 75 (*p* = 0.0212) and 90 (*p* = 0.0032) minutes. However, there was no significant difference in absorption rate between Tariquidar and α-MG (Figure 6). The results demonstrated that α-MG has an ability to increase the Rho123 absorption rate especially at 75 and 90 min demonstrating a significant difference. However, the potency of α-MG in the increasing absorption rate was very low when compared to the specific Pgp inhibitor (Tariquidar, Kd 5.1 nM).

## 3. Discussion

α- and β-MG were predicted to be both Pgp substrates and Pgp inhibitors via the in silico Pgp model in ADMET Predictor^TM^, which is consistent with the reduction in Pgp ATPase activity by both test compounds in the in vitro study. Based on these results, a mechanism of these test compounds to Pgp was forecasted. The possible mechanism was via interaction with the Pgp transporter. This interaction was confirmed by Laksmiani [22], who demonstrated an affinity of α-MG to Pgp by molecular docking. After the interaction, the test compounds inhibited Pgp efflux transport, which appeared as a noncompetitive inhibitor of substrate transport. The possibility of this mechanism came from an in silico model and Pgp ATPase assay that the MG decreased Pgp ATPase activity. In contrast, Verapamil, a competitive Pgp inhibitor, which acts as a positive control in the assay, increased Pgp ATPase activity. From this study, α- and β-MG at nontoxic concentrations acted as a noncompetitive Pgp inhibitor. Furukawa et al. also found a noncompetitive inhibition of α-MG on Ca^2+^-pumping ATPase from skeletal muscle sarcoplasmic reticulum [26].

In vitro assays were evidence towards confirming the prediction. In the transport study, an ER was used as an indicator of active transport. Compounds containing an ER greater than 2.0 involved an efflux mechanism [27]. Rho123, a common Pgp substrate, had an approximate ER value of 66. Both α- and β-MG significantly reduced the ER of Rho123 by altering an active transport mechanism in all treatment groups with a non-significant difference between both compounds. Moreover, the TEER values and LY assays in all transport experiments demonstrated that both compounds did not alter the integrity of Caco-2 cell monolayers and paracellular transport. The MDR1 expression study was conducted to evaluate a regulation in Pgp expression affecting Pgp activity. The result was that α- and β-MG significantly downregulated *MDR1* gene expression, contributing to the reduction in the Pgp transporter protein. Moreover, the mRNA expression result was consistent with the 7-day pre-treatment study, which decreased Pgp activity. Thus, the possible mechanisms of α- and β-MG in inhibiting the Pgp transporter across Caco-2 cells might be a non-competitive inhibition of Pgp by interfering Pgp ATPase activity or binding with Pgp to a non-active mode. Furthermore, it inhibited Pgp activity by downregulating *MDR1* expression, which may result in a reduction in the Pgp transporter protein.

It was known that Caco-2 cell monolayers not only expressed transporter proteins and efflux proteins but also expressed Phase II conjugation enzymes. Chitchumroonchokchai et al. [28] found free and conjugated (glucuronidated/sulfated) xanthones in serum and urine after drinking 60 mL 100% mangosteen juice. Furthermore, The Gutierrez-Orozco et al. study [29] confirmed that enterocyte-like Caco-2 cells metabolized α-MG to Phase II conjugation. Thus, Phase II metabolism and conjugation of the xanthone compounds were another possible factor that affected the inhibition of the Pgp transporter across Caco-2 cells.

The ex vivo absorptive transport study was conducted to investigate actual transport. α-MG was chosen as a test sample because it is a main xanthone compound obtained by extraction from mangosteen pericarp and the results of inhibiting Pgp both in in silico and cell studies showed no significant difference compared to β-MG. Moreover, the amount of β-MG in our lab was very small and was not enough for this experiment. The results from the ex vivo transport study indicated that α-MG had a Pgp affinity inhibitor leading to increased absorption of the Pgp substrate in the serosal side. The difference in the pharmacokinetics of oral administration between Tariquidar and α-MG was further compared. Data were collected from Matzneller et al. [30] and Li et al. [31]. Results showed that the absolute bioavailability (F) in the solution dosage form was 71.6% for Tariquidar at a dosage of 15 mg/kg and 90.2% for α-MG at a dosage of 20 mg/kg. The comparison of pharmacokinetics between Tariquidar and α-MG is detailed in Table S3. Thus, α-MG oral administration in rats expressed a great F when compared to Tariquidar. Furthermore, the authors are in the process of modifying α and β-MG molecules to obtain improved ADMET properties [32] and also to develop formulation/dosage forms to improve F results in the future. In conclusion, the α- and β-MGs were potential candidates for Pgp modifiers to increase the oral bioavailability of Pgp substrate drugs and treat upregulated Pgp cells as in cancer cells.

## 4. Materials and Methods

### 4.1. Source of Extracts

The hulls of *Garcinia mangostana* were collected at Had-Yai Market, Songkhla Province, in southern Thailand. Air-dried hulls of *G. mangostana* were extracted with CH_2_Cl_2_ (2 × 20 L) for 5 days at room temperature. The crude CH_2_Cl_2_ extracts were evaporated under reduced pressure to obtain a brownish natural crude extract, which was subjected to quick column chromatography (QCC) on silica gel (Merck 60 F254) using hexane as a first eluent and then, increasing the polarity with acetone to give 21 fractions (A1–A21). Fraction A3 was further separated by column chromatography (CC) eluting with a gradient of EtOAc-hexane to give 39 subfractions (A3.1–A3.39) and β-mangostin (12.5 mg). Fraction A8 was further separated by CC eluting with a gradient of EtOAc-hexane to give 16 subfractions (A8.1–A8.16) and α-mangostin (336.3 mg), respectively. Ultraviolet-visible (UV-vis), Fourier transform infrared (FTIR), and Nuclear Magnetic Resonance (NMR) spectroscopic techniques were used to analyze the extracts and the spectroscopic data are illustrated in Table S4.

### 4.2. In Silico Study via ADMET Predictor^TM^ Software

ADMET Predictor™ was used first to predict the possibility of xanthone compounds in interaction with Pgp. The molecular structures of mangosteen’s bioactive compounds were drawn in two-dimension via MedChem Designer™ and analyzed via ADMET Predictor™ (version 7.0, Simulation Plus, Lancaster, CA, USA). Two Pgp models were employed in this study: Pgp Substrate and Pgp Inhibitor (*ADMET Predictior^TM^* abbreviations: Pgp_Substr and Pgp_Inh.) The data for Pgp_Substr were taken from the literature [33] and commercial bidirectional Caco-2 permeability assays. The reliability of the literature data was evaluated using criteria similar to those proposed by Zhang et al. [34] and Didziapetris et al. [35]. The dataset was increased to cover a wider chemical space by adding results from a commercial assay. The ADMET Predictor™ development team set the other 122 known Pgp substrates and non-substrates as an external test set. For the Pgp_Inh data, 1214 compounds were collected from the publication of Broccatelli et al. [36], who collected it from the open literature and from the NIHM Psychoactive Drug Screening Program (PDSP). The final dataset contains 620 molecules that inhibit Pgp and 594 molecules that do not.

### 4.3. Pgp ATPase Assay

The Pgp ATPase assay detects the effects of compounds on recombinant human Pgp in a cell membrane fraction. The study depends on the ATP dependence of the light-generating reaction of firefly luciferase. The assay was performed in two steps based on the Pgp-Glo™ assay system [37]. The initial step was the Pgp ATPase reaction. Recombinant human Pgp membrane fractions were incubated with a test compound (TC) and ATP. The second step was ATP detection. The plate was then removed from an incubator and ATP detection reagent was added to stop the reaction and develop a luminescence signal. The luminescence signal corresponds to the remaining unmetabolized ATP, which is represented as relative light units (RLU). The effect of the test compound on Pgp ATPase activity is examined by comparing untreated (NT) samples and samples treated with a TC to a Na_3_VO_4_-treated control.

Basal Pgp ATPase activity (∆*RLU_basal_*) and Pgp ATPase activity in the presence of the TC (∆*RLU_TC_*) were calculated as follows:

(1) $$\Delta RLU_{basal}=RLU_{Na_{3}VO_{4\;}}-RLU_{NT}$$

(2) $$\Delta RLU_{TC}=RLU_{Na_{3}VO_{4\;}}-RLU_{TC}$$

The results were presented as an average of at least three independent experiments.

### 4.4. Cell Culture

Caco-2 cells, purchased from the American Type Culture Collection (ATCC HTB-37 passage 25-35 from original passage number 18, Rockville, MD, USA), were cultured in high glucose Dulbecco’s modified Eagle medium (DMEM) containing 10% fetal bovine serum, 1% nonessential amino acid, and 1% penicillin/streptomycin from Life Technology Gibco. The pH of the medium was adjusted to 7.4. Caco-2 cells were maintained at 37 °C, 5% CO_2_/95% air in a humid environment. In the present study, Caco-2 cells were utilized between passages 28 and 36.

#### 4.4.1. In Vitro Cytotoxicity Studies

Caco-2 cells at a seeding density of 20,000 cells/well were cultured in a 96-well plate. After reaching confluence, the culture medium was replaced by fresh culture medium containing the test compound at a concentration range of 0–100 µM in 0.5% DMSO. Cell viability was determined by the MTT assay after 24 h of incubation. Absorbance was measured at 590 nm using a Spark^®^ 10M microplate reader (TECAN). The results were from at least three independent experiments with triplicate wells. Wells with no cells were used as blanks; wells containing cells with 0.5% DMSO were used as a negative control, and wells containing cells with 2% triton-x 100 were used as a positive control. The results were illustrated as a percentage of cell viability.

#### 4.4.2. Transport Study

Caco-2 cell monolayers were cultured on polycarbonate membrane Transwell^®^ inserts in 24-well plates at a seeding density of 60,000 cells/cm^2^. The experiment started 20 to 21 days post-seeding.

TEER is the measurement of electrical resistance across a Caco-2 cell monolayer in ohmic resistance. For electrical measurements, a chopstick probe was used, a shorter electrode placed in the medium in the upper compartment (apical side) and the longer placed in the medium in the lower compartment (basolateral side). Both electrodes were separated by the cell monolayer and the resistance was measured by a portable voltmeter using a Millicell^®^ ERS (Electrical Resistance System) meter (Millipore, Bedford, MA, USA). TEER measurement was used because it does not damage cells. For our laboratory, a TEER value of 500–1000 Ω cm^2^ was included in the transport study.

The TEER value was calculated as:

(3) TEER values=(R − Rblank) × A

where R is the TEER measurement across a cell monolayer (Ω); Rblank is the TEER measurement of a blank Transwell^®^ insert covered by cell culture media (Ω); A was a surface area of a Transwell^®^ insert filter (cm^2^).

##### Paracellular Transport Study

The effect of co-solvent (0.5% DMSO) and TC on paracellular transport across Caco-2 cell monolayers was evaluated by measuring the TEER value before starting and after finishing an experiment, and LY assay by measuring the passive transport of LY across Caco-2 cell monolayers in A–B direction. Briefly, TC containing 100 µM LY in the transport medium (HBSS with 25 mM HEPES, pH 7.4) was added to an apical compartment, while the blank transport medium was added to a basal compartment. During the transport experiment, the Transwell^®^ plate was placed on an orbital shaker (100 rpm at 37 °C) and sampling withdrawn from a basal compartment was collected to determine the concentration of LY at 60 and 120 min. An amount of LY was measured by a fluorescence plate reader in relative fluorescence units (RFU) using a 428 nm excitation and 540 nm emission.

##### Bidirectional Transport Study

The study was separated into three treatment groups: a co-administration study (co-treated), an inhibition study (pre 30 min), and a 7-day pre-treatment study (pre 7 days).

In this study, 5 µM Rho123 was used as a fluorescence marker and 1 and 5 µM TC in transport medium with 0.5% DMSO were prepared.

In a co-administration study (co-treated), the effect of TC on the Pgp efflux transporter was examined. Rho123 and TC were combined in transport medium and added to a donor compartment. In the receiver compartment, a blank transport medium was added. The transport study was performed in the same conditions as a paracellular study. At 30, 60, 90, and 120 min, a transport medium from a receiver compartment was sampled. A concentration of Rho123 in the medium that transported across Caco-2 monolayers was analyzed by a fluorescence microplate reader at the excitation wavelength at 485 nm and the emission wavelength at 528 nm. An apical-to-basolateral (A–B or absorptive) transport and a basolateral-to-apical (B–A or efflux) transport were studied.

In an inhibition study (pre 30 min), the difference in incubation TC between co-treated with a Pgp substrate and pre-treated 30 min before adding a Pgp substrate was investigated. Cell monolayers were pre-incubated with a TC on both the apical and basolateral side for 30 min prior to adding Rho123. During the experiment, the TC was also presented in both sides at a constant concentration over time until the end of the experiment.

In the 7-day pre-treatment study (pre 7 days), the effect of TC on Pgp protein expression was investigated. TC was pre-incubated on the cell monolayers for 7 days before an experiment. On the experiment day, the TC was removed. Only Rho13 in transport medium was added to a donor compartment.

In all treatment groups, 5 µM Rho123 in transport medium was used as a control and 5 µM Rho123 containing 100 µM verapamil was used as a positive control. TEER and the LY assay were also determined in parallel with transport experiments to verify the integrity of the cell monolayers.

##### Data Analysis

P_app_ and LY Rejection were calculated to confirm any damage of the Caco-2 cell monolayer during the experiment. P_app_ (in cm/s) and %LY Rejection were calculated based on the following formulas.

(4) $$P_{app}=\frac{dQ}{dt}\times \frac{1}{AC_{0}}$$

According to the equation, *dQ*/*dt* was the permeability rate (µmol/s), which is the slope of a plot of the cumulative receiver concentration by time; *A* was the membrane surface area (cm^2^); and *C*_0_ was the initial donor concentration (µmol/mL).

%LY Rejection = 100 × [1 − RFUbasolateral/RFUapical](5)

For the bidirectional transport study, ER was calculated based on the following equation.

(6) $$\mathrm{ER}=\frac{P_{app}\left(B-A\right)}{{\;P}_{app}\;\left(A\;-\;B\right)}$$

#### 4.4.3. *MDR1* Expression Using a Real-Time Reverse Transcription Quantitative Polymerase Chain Reaction (Real Time RT-qPCR)

Total RNA was extracted using a GeneJET RNA Purification Kit (ThermoFisher Scientific) according to the manufacturer’s protocol after exposing 21 days of culturing Caco-2 cells to xanthone compounds for 9 h. The reverse transcription reaction was performed using Kapa SYBR^®^ FAST One-step qRT-PCR Universal (Kapa biosystems, Wilmington, MA, USA) and MxPro-Mx3000P (Agilent, Santa Clara, CA, USA). All sequences of primers were shown in Table 2. Glutaraldehyde-3-phosphate dehydrogenase (GAPDH) was used as an internal control. An expression of *MDR1* was determined in relation to untreated cells. The Ct (cycle threshold) data were automatically collected and calculated to the delta-delta Ct (ΔΔCt) and 2-ΔΔCt in order to analyze the quantitative RT-PCR results.

### 4.5. Ex Vivo Absorptive Transport Study across Everted Mouse Ileum

Experiments with animals were performed at the Laboratory of Drug Delivery Systems, Kobe Gakuin University, Japan in compliance with the regulations of the Committee on Ethics in the Care and Use of Laboratory Animals. Male ddY mice weighing 30 g (6 weeks) were housed in controlled environments between 23 ± 1 °C and 55 ± 5% relative humidity with free access to water and food during acclimatization.

The experiment was performed according to Tomura et al. [38]. Mice were anesthetized with ether and euthanized by exsanguination of an abdominal aorta. An ileum was removed immediately and rinsed in ice-cold Krebs–Ringer–Henseleit bicarbonate buffer (KRB, pH 6.5). The ileum was divided into 2 segments approximately 5 cm long for each segment and everted using a stainless-steel rod. Polyethylene tubes were inserted into both ends and tied. The everted ileum was placed in 40 mL KRB with 5% O_2_/95% CO_2_ at 37 °C and perfused with KRB at 0.1 mL/min using an infusion pump during the experiment. Rho123 solution (a marker for Pgp function) was added to a mucosal side to obtain a final concentration of 10 μM. Either positive control tariquidar or α-MG was co-administered with Rho123 to obtain a final concentration of 100 nM for tariquidar or 200 μM for α-MG. α-MG was dissolved in 10% ethanol/10% tween80 for stock solution and 0.1% ethanol/0.1%tween80 for final concentration in the everted sac experiment. Under bubbling O_2_/CO_2_ gas, the transport of Rho123 from the mucosal to serosal side was studied in controlled conditions, and the presence of the inhibitor or the presence of α-MG were evaluated. The outflow was collected for 5 min at 15 min intervals for up to 90 min. Rho123 concentration in the outflow was determined by a microplate fluorescence reader at 485 nm excitation and 528 nm emission. The absorption rate of Rho123 in the outflow was calculated according to the following equation:

(7) $$Absortion\;rate=\;\frac{\left(Rho123\;conc.\;in\;outflow\times perfusion\;rate\right)}{length\;of\;ilem\;segment}$$

### 4.6. Statistical Methods

Data were presented as means ± SE from triplicate measurements in at least three experiments. Statistical analysis of the data was performed using ANOVA. The statistical tests were carried out using SPSS 23.0 software and a *p*-value < 0.05 was considered to be statistically significant.

## Figures and Tables

**Table 1:** The predictive likelihood of α-mangostin (α-MG) and β-mangostin (β-MG) to interact with Pgp via the ADMET Predictor™ program.

Properties	α-MG	β-MG
Likelihood of Pgp Inhibitor (%confidence score)	Yes (97%)	Yes (97%)
Likelihood of Pgp Substrate (%confidence score)	Yes (68%)	Yes (95%)

**Table 2:** Primer gene sequences for real-time RT-qPCR.

Primer		Gene Sequences
*MDR1*	Forward Reverse	5′TGC TCA GAC AGG ATG TGA GTT G 3′ 5′AAT TAC AGC AAG CCT GGA ACC 3′
GAPDH	Forward Reverse	5′GGC CTC CAA GGA GTA AGA CC 3′ 5′AGG GGA GAT TCA GTG TGG TG 3′

## Supplementary Materials

The following are available online. Figure S1: The TEER value at seeding density 60,000 cells/cm^2^ (a) TEER value measure every other day before changing cell media from day 1-21; (b) TEER value before start bidirectional transport experiment and after finish the experiment. Data are presented as mean ± SE from triplicate measurements in all experiments (co-treated, pre-treated 30 mins, and pre 7 days 40 with α-MG or β-MG); Table S1: The apical-to-basal permeability coefficients (Papp) of Lucifer Yellow (LY) Permeability Assay and Percent LY rejection in each condition; Table S2: The Permeability coefficient (Papp) and ER of Rho123 when treated with α-MG or β-MG; Table S3: Comparison of pharmacokinetics between Tariquidar and α-MG (oral absorption in rats, solution dosage form); Table S4: Spectroscopic data of α- and β-mangostin.
