# Supplementary material for: The Intestinal Efflux Transporter Inhibition Activity of Xanthones from Mangosteen Pericarp: An In Silico, In Vitro and Ex Vivo Approach
Source: Molecules. 2020 Dec 11;25(24):5877. doi: 10.3390/molecules25245877 (PMC7764676; doi:10.3390/molecules25245877)
Supplement: Supplementary file 1 [file molecules-25-05877-s001.pdf]

molecules

MDPI

Supporting information

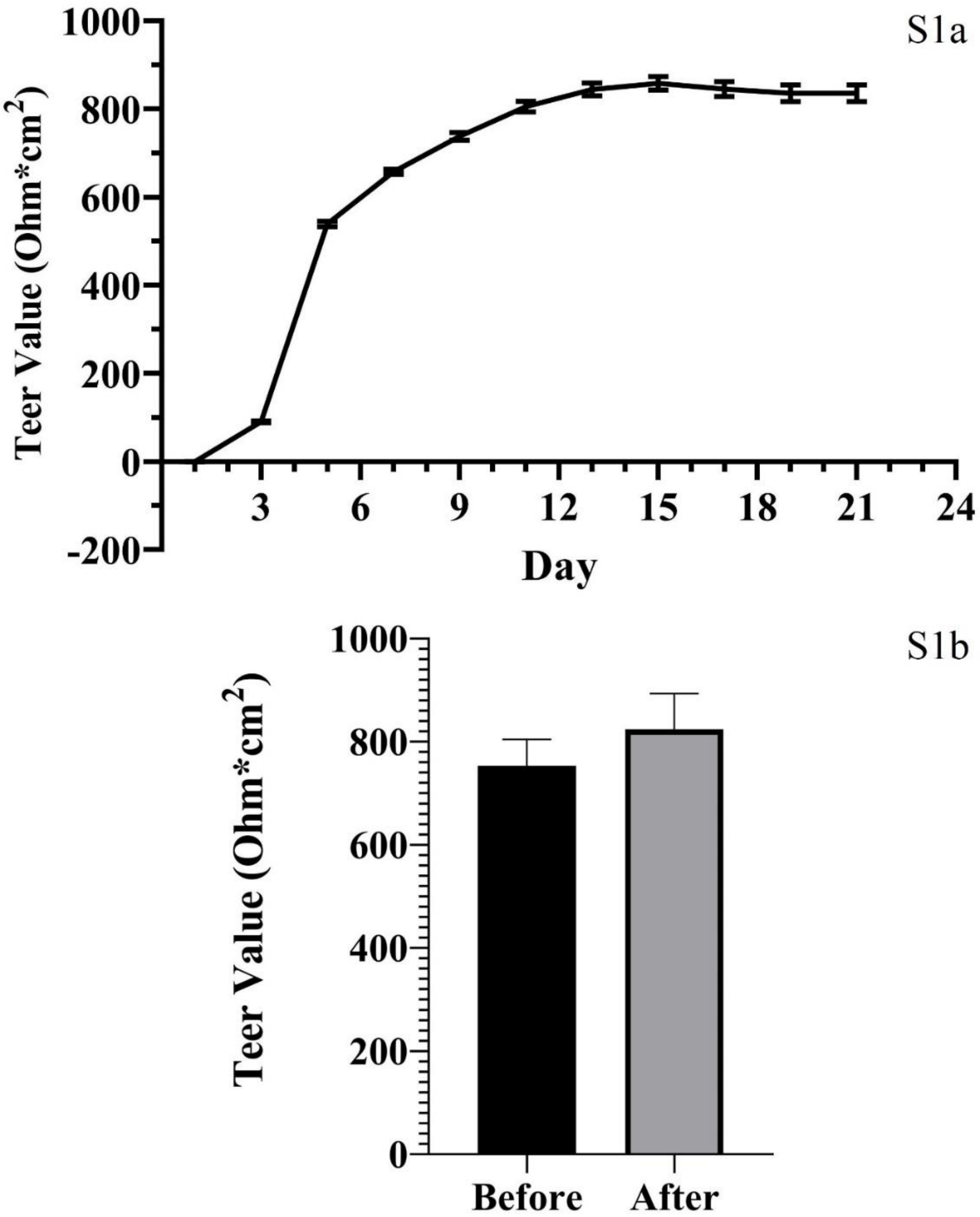

Figure S1. The TEER value at seeding density 60,000 cells/cm^2 (a, TEER value measure every other day before changing cell media from day 1-21; b, TEER value before start bidirectional transport experiment and after finish the experiment. Data are presented as mean ± SE from triplicate measurements in all experiments (co-treated, pre-treated 30 mins, and pre 7 days with α-MG or β-MG).

molecules

MDPI

Table S1. The apical-to-basal permeability coefficients (Papp) of Lucifer Yellow (LY) Permeability Assay and Percent LY rejection in each condition.

Condition	% LY rejection	P_{app} (cm/sec) x 10^{-7}
- DMSO 60,000 cells/cm^2	99.87 ± 0.0010	1.994 ± 0.020
+ DMSO 60,000 cells/cm^2	99.90 ± 0.0003	1.375 ± 0.023
+ α-MG 5 μM 60,000 cells/cm^2	99.82 ± 0.0464	1.396 ± 0.075
+ α-MG 1 μM 60,000 cells/cm^2	99.89 ± 0.0007	1.530 ± 0.012
+ β-MG 5 μM 60,000 cells/cm^2	99.87 ± 0.0374	1.311 ± 0.022
+ β-MG 1 μM 60,000 cells/cm^2	99.93 ± 0.0015	1.013 ± 0.013

Data are presented as mean ± SE from triplicate measurements in four experiments.

Table S2. The Permeability coefficient (P_{app}) and ER of Rho123 when treated with α-MG or β-MG.

Experiment	P_{app} A-B (cm/sec) x 10^{-7}	P_{app} B-A (cm/sec) x 10^{-5}	ER
**Co-treated**			
Rho123 (Control)	1.101 ± 0.070	0.699 ± 0.093	66.26 ± 0.00
+ α-MG 5 μM	1.294 ± 0.165	*0.496 ± 0.086	***44.68 ± 2.93
+ α-MG 1 μM	0.955 ±0.010	***0.373 ± 0.013	58.21 ± 1.66
+ β-MG 5 μM	1.323 ± 0.214	*0.476 ± 0.097	***46.08 ± 5.74
+ β-MG 1 μM	1.408 ± 0.339	0.563 ± 0.174	*48.81 ± 6.33
+ Verapamil 100 μM	***1.805 ± 0.131	***0.042 ± 0.005	***2.34 ± 0.39
**Pre-treated 30 min**			
Rho123 (Control)	1.313 ± 0.001	1.019 ± 0.002	77.57 ± 0.17
+ α-MG 5 μM	***1.191 ± 0.017	***0.558 ± 0.003	***46.86 ± 0.40
+ α-MG 1 μM	***1.794 ± 0.012	***0.872 ± 0.002	***48.59 ± 0.41
+ β-MG 5 μM	***1.191 ± 0.017	***0.466 ± 0.003	***39.14 ± 0.67
+ β-MG 1 μM	***1.521 ± 0.032	***0.814 ± 0.013	***53.54 ± 0.36
+ Verapamil 100 μM	***1.727 ± 0.024	***0.041 ± 0.000	***2.39 ± 0.04
**Pre-treated 7 days**			
Rho123 (Control)	1.274 ± 0.017	0.968 ± 0.022	75.93 ± 0.74
+ α-MG 5 μM	***1.883 ± 0.108	***0.777 ± 0.009	***42.10 ± 2.88
+ β-MG 5 μM	***1.882 ± 0.141	***0.740 ± 0.004	***40.55 ± 3.25
+ Verapamil 100 μM	***1.961 ± 0.116	***0.059 ± 0.003	***3.10 ± 0.32

Data presented as mean ± SE (* p<0.05, *** p<0.001 compared to the control)

molecules

Table S3. Comparison of pharmacokinetics between Tariquidar and α-MG (oral absorption in rats, solution dosage form)

Topics	Tariquidar [14]	α-MG [15]
Dose	15 mg/kg in ≤ 2% DMSO	20 mg/kg in 2% ethanol and 2% Tween 80
C_{max}	1.2 μg/mL	Let use C_0 as approximation(a): 10.35 μg/mL (if same dose as tariquidar 15/20 * 10.35 = 7.76 μg/mL)
T_{max}	4.0 h	Calculate it is rapidly absorbed approx.(b) 0.373 h.
AUC	18.1 μg*h/mL	1.237 μg*h/mL
F (%)	Absolute 71.6%	Absolute calculated $AUC_{oral/IV}$ = 1.237/1.372 *100 = 90.2%

(a) Let simple use C at time 0 for approximation.
(b) Approximate by one compartment oral $t_{max} = \ln(k_a/k_e)/(k_a-k_e)$, k_a = k10 = 8.24 hr^{-1}, k_e = CL/Vp = 1.70/3.76 = 0.452 hr^{-1}, Thus tmax = ln (8.24/0.452)/(8.24-0.452) = 0.373 h

Table S4. Spectroscopic data of α- and β-mangostin

Compounds	Properties	Spectroscopic properties		
		UV-Vis ($CHCl_3$)	FT-IR (neat)	^{1}H NMR 300 MHz ($CDCl_3$) δ (ppm)
α-mangostin	Deep-yellow powder, m.p. 180-182 °C	λ_{max} = 209, 244, 261, 317, 368 nm	ν_{max} 3306, 1642 cm^{-1}	13.77 (1H, s, 1-OH), 6.29 (1H, s, H-4), 6.82 (1H, s, H-5), 3.45 (2H, d, J = 7.2 Hz, H-1′), 5.29 (1H, m, H-2′), 1.77 (1H, s, H-4′), 1.84 (1H, s, H-5′), 4.09 (2H, d, J = 6.0 Hz, H-1″), 5.26 (1H, m, H-2″), 1.69 (3H, s, H-4″), 1.84 (3H, s, H-5″), 3.81 (3H, s, 7-OCH_3)
β-mangostin	Yellow needle-single crystals, m.p. 172-174 °C	λ_{max} = 243, 289, 298, 320, 351, 391 nm	ν_{max} 3397, 1649, 1613 cm^{-1}	13.41 (1H, s, 1-OH), 6.32 (1H, s, H-4), 6.82 (1H, s, H-5), 3.35 (2H, d, J = 7.2 Hz, H-1′), 5.23 (1H, m, H-2′), 1.68 (3H, s, H-4′), 1.80 (3H, s, H-5′), 4.09 (2H, d, J = 7.2 Hz, H-1″), 5.26 (1H, m, H-2″), 1.68 (3H, s, H-4″), 1.83 (3H, s, H-5″), 3.90 (3H, s, 3-OCH_3), 3.81 (3H, s, 7-OCH_3)
